# *catena*-Poly[bis­(μ_3_-2-phenyl­acetato-κ^3^ *O*,*O*′:*O*)bis­(μ_2_-2-phenyl­acetato-κ^2^ *O*:*O*′)dicopper(II)(*Cu*—*Cu*)]

**DOI:** 10.1107/S160053681301581X

**Published:** 2013-06-15

**Authors:** Meriem Benslimane, Yasmine Kheira Redjel, Hocine Merazig, Jean-Claude Daran

**Affiliations:** a Unité de Recherche de Chimie de l’Environnement et Moléculaire Structurale, Faculté des Sciences Exactes, Département de Chimie, Université de Constantine 1, 25000 Constantine, Algeria; b Laboratoire de Chimie de Coordination, UPR-CNRS 8241, 205 route de Narbonne, 31077 Toulouse Cedex 4, France

## Abstract

The title polymeric compound, [Cu_2_(C_8_H_7_O_2_)_4_]_*n*_, was synthesized by the reaction of copper acetate with aqueous phenyl­acetic acid. The unique Cu^II^ atom is coordinated by five O atoms from the carboxyl­ate groups of phenyl­acetate ligands, and the strongly distorted octa­hedral coordination environment is completed by a Cu—Cu bond of 2.581 (2) Å, at whose mid-point is located an inversion centre. The crystal structure consists of infinite polymeric linear chains of Cu^2+^ ions, running along [100], linked by bridging phenyl­acetate groups.

## Related literature

For the biological activity of divalent transition metals, see: Stem *et al.* (1990 ▶); Kimura (1994 ▶). For related compounds, see: Cui *et al.* (1999 ▶); Kong *et al.* (2005*a* ▶,*b* ▶). 
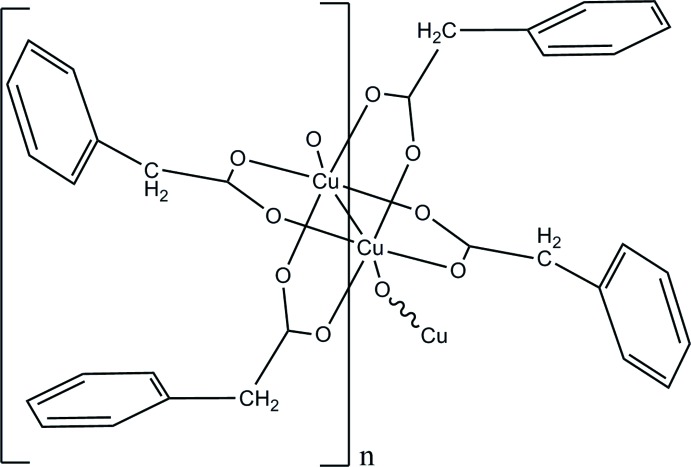


## Experimental

### 

#### Crystal data

[Cu_2_(C_8_H_7_O_2_)_4_]
*M* *_r_* = 667.62
Monoclinic, 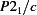

*a* = 5.1829 (6) Å
*b* = 26.328 (4) Å
*c* = 10.2279 (13) Å
β = 97.892 (7)°
*V* = 1382.4 (3) Å^3^
*Z* = 2
Mo *K*α radiation
μ = 1.59 mm^−1^
*T* = 180 K
0.15 × 0.10 × 0.01 mm

#### Data collection

Bruker APEXII diffractometer
Absorption correction: multi-scan (*SADABS*; Bruker, 2008 ▶) *T*_min_ = 0.552, *T*_max_ = 0.745
4056 measured reflections
2382 independent reflections
1927 reflections with *I* > 2σ(*I*)
*R*_int_ = 0.036

#### Refinement

*R*[*F* ^2^ > 2σ(*F* ^2^)] = 0.082
*wR*(*F* ^2^) = 0.254
*S* = 1.20
2382 reflections
190 parameters
H-atom parameters constrained
Δρ_max_ = 2.24 e Å^−3^
Δρ_min_ = −1.14 e Å^−3^

### 

Data collection: *APEX2* (Bruker, 2012 ▶); cell refinement: *SAINT* (Bruker, 2012 ▶); data reduction: *SAINT*; program(s) used to solve structure: *SIR92* (Altomare *et al.*, 1993 ▶); program(s) used to refine structure: *SHELXL97* (Sheldrick, 2008 ▶); molecular graphics: *ORTEPIII* (Burnett & Johnson, 1996 ▶) and *ORTEP-3 for Windows* (Farrugia, 2012 ▶); software used to prepare material for publication: *SHELXL97*.

## Supplementary Material

Crystal structure: contains datablock(s) global, I. DOI: 10.1107/S160053681301581X/lr2106sup1.cif

Structure factors: contains datablock(s) I. DOI: 10.1107/S160053681301581X/lr2106Isup2.hkl

Additional supplementary materials:  crystallographic information; 3D view; checkCIF report

## Figures and Tables

**Fig. 1.**
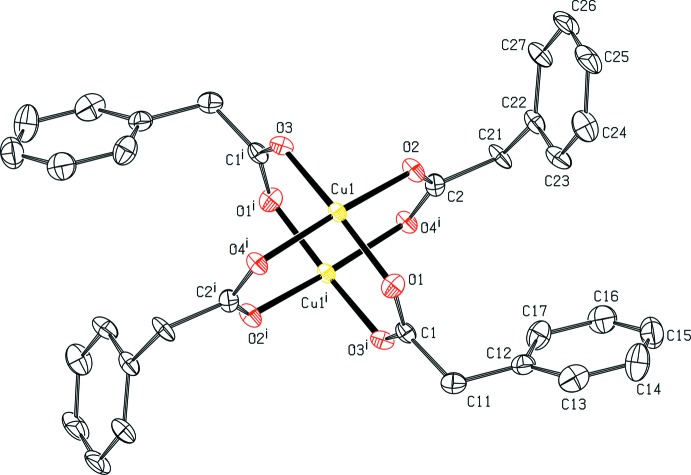
The molecular structure of the title compound. Displacement ellipsoids are drawn at the 50% probability level and H atoms are shown as small spheres of arbitrary radii.[Symmetry code: (i): 2 - x, -y, 1 - z].

**Fig. 2.**
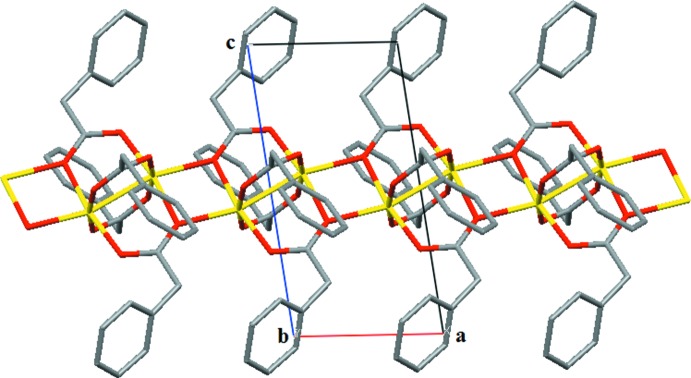
A view of the crystal structure, showing chains along [100]. Hydrogen atoms have been omitted for clarity

## supplementary crystallographic information

### Comment

Carboxylate groups may interact as bridging ligands with divalent transition metals present in the biological environment, thereby altering the bioavailability of the drug. Moreover, it is well known that many complexes of divalent transition metals are capable of catalyzing the hydrolysis of RNA (Stem *et al.*, 1990; Kimura, 1994). The coordination chemistry of polynuclear Cu^2+^ complexes bridged by phenylacetate has not been much reported. To date, we have found only two report of a dinuclear Co^2+^ complexes, namely tetrakis(phenylacetato)bis[(quinoline-*N*)-cobalt(II)] (Cui *et al.*, 1999), *µ*-Aqua-*κ^2^O:O*-di-*µ*-phenylacetato-*κ^4^O:O'* -bis[(1,10-phenanthroline-*κ^2^N,N'*)(phenylacetato- *κO*)cobalt(II)](Kong *et al.*, 2005*a*) and dinuclear Cu^2+^ complex, namely tetrakis(phenylacetato)bis-[(*N*,*N*-dimethylformamide)copper(II)] (Kong *et al.*, 2005*b*), in which all phenylacetate groups are in bidendate bridging modes. In this paper, we describe the crystal structure of new polymeric complex obtained by reaction of phenylacetic acid with copper(II) acetate.

The molecular geometry of the title compound is illustrated in Fig.1. Each Cu^II^ atom is six-coordinated by five O atoms from carboxylate groups of the phenylacetate and is completed by a Cu—Cu bond in a strongly distorted octahedral coordination, in which an inversion center is located at the mid-point of the Cu—Cu bond with a Cu···Cu distance is 2.581 (2) Å. The Cu—O bond length ranges from 1.944 (7) to 2.200 (6) Å. The two carboxylate groups [O3/C1/O1 and O2/C2/O4] are almost perpendicular to one another with a dihedral angle of 78.4 (16)°. The structure, consists of polymeric infinite linear chains running along [100](Fig.2). The chains are formed by Cu^2+^ ions linked with bridging phenylacetate groups.

### Experimental

To a solution of Cu(CH_3_CO_2_)_2_.H_2_O (0.049 g, 0.25 mmol) in methanol (10 cm^3^) at room temperature was added solid phenylacetic acid (0.068 g, 0.5 mmol) in small portions under constant stirring.The mixture was then filtered and the filtrate allowed to stand for 10 days, after which small blue block-like crystals of the title complex were obtained.

### Refinement

The C-bound hydrogen atoms were placed in geometrically idealized positions and constrained to ride on their parent atom positions with a C–H distances of 0.93 Å and with *U*_iso_(H) = 1.2*Ueq*(C).

### Crystal data

**Table:**

[Cu_2_(C_8_H_7_O_2_)_4_]	*F*(000) = 684
*M**_r_* = 667.62	*D*_x_ = 1.604 Mg m^−^^3^
Monoclinic, *P*2_1_/*c*	Mo *K*α radiation, λ = 0.71073 Å
Hall symbol: -P 2ybc	Cell parameters from 4583 reflections
*a* = 5.1829 (6) Å	θ = 3.1–26.3°
*b* = 26.328 (4) Å	µ = 1.59 mm^−^^1^
*c* = 10.2279 (13) Å	*T* = 180 K
β = 97.892 (7)°	Box, blue
*V* = 1382.4 (3) Å^3^	0.15 × 0.1 × 0.01 mm
*Z* = 2	

### Data collection

**Table:**

Bruker APEXII diffractometer	2382 independent reflections
Radiation source: fine-focus sealed tube	1927 reflections with *I* > 2σ(*I*)
Graphite monochromator	*R*_int_ = 0.036
ω and φ scans	θ_max_ = 25.0°, θ_min_ = 2.2°
Absorption correction: multi-scan (*SADABS*; Bruker, 2008)	*h* = −6→6
*T*_min_ = 0.552, *T*_max_ = 0.745	*k* = −22→31
4056 measured reflections	*l* = 0→12

### Refinement

**Table:**

Refinement on *F*^2^	0 restraints
Least-squares matrix: full	H-atom parameters constrained
*R*[*F*^2^ > 2σ(*F*^2^)] = 0.082	*w* = 1/[σ^2^(*F*_o_^2^) + (0.089*P*)^2^ + 25.7899*P*] where *P* = (*F*_o_^2^ + 2*F*_c_^2^)/3
*wR*(*F*^2^) = 0.254	(Δ/σ)_max_ = 0.003
*S* = 1.20	Δρ_max_ = 2.24 e Å^−^^3^
2382 reflections	Δρ_min_ = −1.14 e Å^−^^3^
190 parameters	

### Special details

**Table:**

Geometry. All e.s.d.'s (except the e.s.d. in the dihedral angle between two l.s. planes) are estimated using the full covariance matrix. The cell e.s.d.'s are taken into account individually in the estimation of e.s.d.'s in distances, angles and torsion angles; correlations between e.s.d.'s in cell parameters are only used when they are defined by crystal symmetry. An approximate (isotropic) treatment of cell e.s.d.'s is used for estimating e.s.d.'s involving l.s. planes.
Refinement. Refinement of *F*^2^ against ALL reflections. The weighted *R*-factor *wR* and goodness of fit *S* are based on *F*^2^, conventional *R*-factors *R* are based on *F*, with *F* set to zero for negative *F*^2^. The threshold expression of *F*^2^ > σ(*F*^2^) is used only for calculating *R*-factors(gt) *etc*. and is not relevant to the choice of reflections for refinement. *R*-factors based on *F*^2^ are statistically about twice as large as those based on *F*, and *R*- factors based on ALL data will be even larger.

### Fractional atomic coordinates and isotropic or equivalent isotropic displacement parameters (Å^2^)

**Table:**

	*x*	*y*	*z*	*U*_iso_*/*U*_eq_	
C1	0.9678 (18)	0.0897 (3)	0.4136 (9)	0.017 (2)	
C2	0.8188 (19)	0.0341 (3)	0.7030 (10)	0.019 (2)	
C11	0.948 (2)	0.1445 (4)	0.3711 (10)	0.024 (2)	
H11A	0.8356	0.147	0.2874	0.028*	
H11B	1.1195	0.1567	0.3582	0.028*	
C12	0.840 (2)	0.1779 (4)	0.4725 (10)	0.023 (2)	
C13	0.932 (2)	0.2267 (4)	0.4983 (12)	0.033 (3)	
H13	1.0631	0.2391	0.4536	0.04*	
C14	0.833 (3)	0.2573 (5)	0.5886 (14)	0.045 (3)	
H14	0.8991	0.2898	0.6054	0.054*	
C15	0.634 (3)	0.2395 (5)	0.6546 (12)	0.038 (3)	
H15	0.5646	0.26	0.715	0.046*	
C16	0.542 (2)	0.1914 (5)	0.6295 (12)	0.035 (3)	
H16	0.4109	0.1791	0.6745	0.042*	
C17	0.640 (2)	0.1606 (4)	0.5389 (12)	0.030 (2)	
H17	0.5713	0.1282	0.5219	0.036*	
C21	0.7117 (19)	0.0600 (4)	0.8179 (9)	0.021 (2)	
H21A	0.585	0.0854	0.7834	0.025*	
H21B	0.6227	0.0349	0.865	0.025*	
C22	0.9225 (19)	0.0851 (4)	0.9136 (10)	0.020 (2)	
C23	1.054 (2)	0.1279 (4)	0.8743 (10)	0.025 (2)	
H23	0.9996	0.1426	0.7924	0.03*	
C24	1.265 (2)	0.1486 (4)	0.9567 (11)	0.032 (3)	
H24	1.3534	0.1765	0.9293	0.038*	
C25	1.340 (2)	0.1274 (5)	1.0781 (11)	0.034 (3)	
H25	1.4792	0.1414	1.1332	0.041*	
C26	1.213 (2)	0.0860 (5)	1.1204 (11)	0.033 (3)	
H26	1.2689	0.0717	1.2026	0.04*	
C27	0.998 (2)	0.0652 (4)	1.0378 (10)	0.026 (2)	
H27	0.9073	0.038	1.0672	0.031*	
O1	1.1763 (13)	0.0758 (2)	0.4846 (7)	0.0213 (15)	
O2	1.0628 (12)	0.0308 (3)	0.7092 (6)	0.0193 (15)	
O3	1.2268 (13)	−0.0615 (3)	0.6233 (6)	0.0206 (15)	
O4	1.3446 (11)	−0.0181 (2)	0.3911 (6)	0.0170 (14)	
Cu1	1.2319 (2)	0.00810 (4)	0.56015 (11)	0.0143 (4)	

### Atomic displacement parameters (Å^2^)

**Table:**

	*U*^11^	*U*^22^	*U*^33^	*U*^12^	*U*^13^	*U*^23^
C1	0.016 (5)	0.023 (5)	0.014 (5)	−0.003 (4)	0.008 (4)	−0.001 (4)
C2	0.021 (5)	0.017 (5)	0.021 (5)	−0.003 (4)	0.008 (4)	−0.001 (4)
C11	0.023 (5)	0.028 (5)	0.020 (5)	0.001 (4)	0.002 (4)	0.003 (4)
C12	0.025 (6)	0.024 (5)	0.019 (5)	0.001 (4)	−0.002 (4)	0.002 (4)
C13	0.026 (6)	0.032 (6)	0.040 (7)	−0.003 (5)	−0.001 (5)	0.004 (5)
C14	0.049 (8)	0.027 (6)	0.059 (9)	−0.004 (6)	0.010 (7)	−0.013 (6)
C15	0.039 (7)	0.040 (7)	0.035 (7)	0.008 (5)	0.004 (6)	−0.011 (5)
C16	0.026 (6)	0.044 (7)	0.035 (7)	0.008 (5)	0.012 (5)	0.004 (5)
C17	0.027 (6)	0.022 (5)	0.041 (7)	−0.003 (4)	0.002 (5)	−0.003 (5)
C21	0.013 (5)	0.040 (6)	0.010 (5)	−0.001 (4)	0.003 (4)	−0.009 (4)
C22	0.011 (5)	0.034 (5)	0.016 (5)	0.002 (4)	0.000 (4)	−0.008 (4)
C23	0.023 (5)	0.040 (6)	0.013 (5)	0.000 (4)	0.001 (4)	−0.004 (4)
C24	0.026 (6)	0.037 (6)	0.032 (6)	−0.011 (5)	0.001 (5)	−0.012 (5)
C25	0.030 (6)	0.050 (7)	0.021 (6)	−0.002 (5)	−0.007 (5)	−0.016 (5)
C26	0.035 (7)	0.047 (7)	0.013 (5)	0.005 (5)	−0.007 (5)	−0.010 (5)
C27	0.028 (6)	0.036 (6)	0.015 (5)	−0.002 (5)	0.008 (5)	−0.004 (4)
O1	0.014 (3)	0.023 (3)	0.027 (4)	0.000 (3)	0.001 (3)	0.001 (3)
O2	0.006 (3)	0.035 (4)	0.017 (3)	0.002 (3)	0.001 (3)	−0.006 (3)
O3	0.023 (4)	0.025 (4)	0.014 (3)	0.000 (3)	0.002 (3)	0.002 (3)
O4	0.005 (3)	0.030 (4)	0.016 (3)	0.000 (3)	0.002 (3)	−0.005 (3)
Cu1	0.0114 (6)	0.0186 (6)	0.0134 (6)	−0.0004 (4)	0.0033 (4)	−0.0023 (5)

### Geometric parameters (Å, º)

**Table:**

C1—O3^i^	1.267 (11)	C21—H21B	0.97
C1—O1	1.270 (12)	C22—C27	1.380 (15)
C1—C11	1.507 (13)	C22—C23	1.402 (15)
C2—O2	1.261 (12)	C23—C24	1.397 (14)
C2—O4^i^	1.264 (12)	C23—H23	0.93
C2—C21	1.527 (13)	C24—C25	1.368 (16)
C11—C12	1.524 (14)	C24—H24	0.93
C11—H11A	0.97	C25—C26	1.371 (17)
C11—H11B	0.97	C25—H25	0.93
C12—C13	1.381 (15)	C26—C27	1.414 (15)
C12—C17	1.395 (15)	C26—H26	0.93
C13—C14	1.377 (17)	C27—H27	0.93
C13—H13	0.93	O1—Cu1	1.948 (7)
C14—C15	1.388 (19)	O2—Cu1	1.954 (6)
C14—H14	0.93	O3—C1^i^	1.267 (11)
C15—C16	1.365 (17)	O3—Cu1	1.944 (7)
C15—H15	0.93	O4—C2^i^	1.264 (12)
C16—C17	1.379 (16)	O4—Cu1	2.021 (6)
C16—H16	0.93	O4—Cu1^ii^	2.199 (6)
C17—H17	0.93	Cu1—O4^ii^	2.199 (6)
C21—C22	1.515 (13)	Cu1—Cu1^i^	2.581 (2)
C21—H21A	0.97		
			
O3^i^—C1—O1	125.6 (9)	C23—C22—C21	120.0 (9)
O3^i^—C1—C11	117.1 (9)	C24—C23—C22	120.7 (10)
O1—C1—C11	117.3 (8)	C24—C23—H23	119.6
O2—C2—O4^i^	125.3 (9)	C22—C23—H23	119.6
O2—C2—C21	117.4 (9)	C25—C24—C23	119.3 (11)
O4^i^—C2—C21	117.3 (8)	C25—C24—H24	120.4
C1—C11—C12	111.9 (8)	C23—C24—H24	120.4
C1—C11—H11A	109.2	C24—C25—C26	121.5 (10)
C12—C11—H11A	109.2	C24—C25—H25	119.3
C1—C11—H11B	109.2	C26—C25—H25	119.3
C12—C11—H11B	109.2	C25—C26—C27	119.4 (10)
H11A—C11—H11B	107.9	C25—C26—H26	120.3
C13—C12—C17	118.0 (10)	C27—C26—H26	120.3
C13—C12—C11	121.2 (10)	C22—C27—C26	120.3 (10)
C17—C12—C11	120.7 (9)	C22—C27—H27	119.9
C14—C13—C12	121.5 (11)	C26—C27—H27	119.9
C14—C13—H13	119.3	C1—O1—Cu1	123.9 (6)
C12—C13—H13	119.3	C2—O2—Cu1	122.4 (6)
C13—C14—C15	119.9 (11)	C1^i^—O3—Cu1	119.9 (6)
C13—C14—H14	120	C2^i^—O4—Cu1	121.6 (6)
C15—C14—H14	120	C2^i^—O4—Cu1^ii^	139.1 (6)
C16—C15—C14	119.0 (11)	Cu1—O4—Cu1^ii^	99.3 (3)
C16—C15—H15	120.5	O3—Cu1—O1	170.4 (3)
C14—C15—H15	120.5	O3—Cu1—O2	90.1 (3)
C15—C16—C17	121.4 (11)	O1—Cu1—O2	88.4 (3)
C15—C16—H16	119.3	O3—Cu1—O4	88.9 (3)
C17—C16—H16	119.3	O1—Cu1—O4	91.0 (3)
C16—C17—C12	120.1 (10)	O2—Cu1—O4	170.2 (3)
C16—C17—H17	119.9	O3—Cu1—O4^ii^	95.5 (3)
C12—C17—H17	119.9	O1—Cu1—O4^ii^	93.9 (3)
C22—C21—C2	112.7 (8)	O2—Cu1—O4^ii^	109.1 (3)
C22—C21—H21A	109.1	O4—Cu1—O4^ii^	80.7 (3)
C2—C21—H21A	109.1	O3—Cu1—Cu1^i^	87.0 (2)
C22—C21—H21B	109.1	O1—Cu1—Cu1^i^	83.4 (2)
C2—C21—H21B	109.1	O2—Cu1—Cu1^i^	86.24 (19)
H21A—C21—H21B	107.8	O4—Cu1—Cu1^i^	83.99 (18)
C27—C22—C23	118.8 (9)	O4^ii^—Cu1—Cu1^i^	164.41 (18)
C27—C22—C21	121.1 (9)		

Symmetry codes: (i) −*x*+2, −*y*, −*z*+1; (ii) −*x*+3, −*y*, −*z*+1.
